# Advances in Mesenchymal Stem Cell Research Applications for Female Infertility-Mechanisms, Efficacy Parameters, Challenges and Future Roadmap

**DOI:** 10.31661/gmj.v13i.3632

**Published:** 2024-10-08

**Authors:** Sina Vakili, Morteza Jafarinia

**Affiliations:** ^1^ Infertility Research Centre, Shiraz University of Medical Sciences, Shiraz, Iran; ^2^ Shiraz Neuroscience Research Center, Shiraz University of Medical Sciences, Shiraz, Iran

**Keywords:** Mesenchymal Stem Cells, Infertility, Premature Ovarian Failure, Polycystic Ovary Syndrome, Endometriosis

## Abstract

Infertility affects approximately 15-20% of couples globally, with female factors contributing to nearly half of cases. Conditions such as polycystic ovary syndrome, endometriosis, tubal damage and premature ovarian failure are leading causes of female infertility. Current treatments like in vitro fertilization (IVF) have limitations and risks. Mesenchymal stem cells (MSCs) have shown therapeutic potential due to their ability to differentiate, secrete trophic factors, and exhibit immunomodulatory and anti-inflammatory properties. They have been demonstrated to repair and regenerate reproductive organs in various preclinical models of infertility related conditions. MSCs have reduced endometriotic lesions, regenerated lost follicles in premature ovarian failure (POF) models, and promoted tubal repair in damage models. Some clinical and preclinical studies have reported improved outcomes with MSC therapy in endometriosis and premature ovarian failure patients. This review discusses the properties and sources of MSCs, their mechanisms of action, preclinical evidence for applications in conditions like POF, polycystic ovary syndrome (PCOS), endometriosis, Asherman syndrome, and preeclampsia, and preliminary clinical data on MSC therapy for female infertility management.

## Introduction

Infertility is a significant global health issue that affects approximately 15% to 20% of couples, with female factors contributing to nearly half of these cases. Defined as the inability to achieve a successful pregnancy after 12 months of unprotected intercourse, female infertility can arise from a myriad of causes, including hormonal imbalances, structural abnormalities in the reproductive organs, and genetic factors. Conditions such as polycystic ovary syndrome (PCOS), endometriosis, and uterine fibroids are prevalent among women facing infertility challenges [1]. The complexity of female infertility necessitates a comprehensive understanding of its underlying causes, which can range from anatomical issues, such as blocked fallopian tubes or uterine abnormalities,to endocrine disorders that disrupt ovulation [2]. Moreover, age plays a pivotal role; the quality and quantity of a woman’s oocytes decline significantly with advancing age, further complicating the landscape of infertility treatment [3]. The importance of innovative treatments in addressing female infertility cannot be overstated. Traditional approaches, including hormonal therapies and assisted reproductive technologies (ART) like in vitro fertilization (IVF), have provided avenues for many couples to conceive. However, these methods often come with limitations such as high costs, invasive procedures, and varying success rates [4]. As such, there is an urgent need for alternative therapies that can enhance fertility outcomes while minimizing physical and emotional burdens on women. Recent advancements in regenerative medicine, particularly the use of mesenchymal stem cells (MSCs), present promising new horizons in the treatment landscape for female infertility [5].

MSCs possess unique properties that make them particularly suitable for addressing reproductive health issues [6]. They can differentiate into various cell types and have strong immunomodulatory effects, which may help restore normal ovarian function and improve uterine receptivity [7]. Research into MSCs has shown potential benefits in treating conditions like ovarian insufficiency and endometrial dysfunction, which are significant contributors to infertility [8]. Additionally, MSCs can be sourced from various tissues—such as bone marrow, adipose tissue, and umbilical cord blood—making them relatively accessible for therapeutic applications [9]. The exploration of MSCs as a treatment modality aligns with the broader trend towards personalized medicine in reproductive health [10]. By harnessing the regenerative capabilities of stem cells, clinicians may offer more tailored approaches to individual patients based on their specific conditions and needs. This shift not only aims to improve fertility outcomes but also seeks to enhance overall reproductive health by addressing underlying pathologies that contribute to infertility.

Furthermore, innovative treatments like MSC therapy could alleviate some of the psychological distress associated with infertility. The emotional toll of unsuccessful attempts at conception can lead to anxiety and depression among affected women [11]. By expanding the arsenal of available treatments and improving success rates, innovative therapies may provide hope and relief for many couples facing the challenges of infertility.

## Mechanisms of Action of MSCs

MSCs are multipotent adult stem cells characterized by their remarkable biological properties, which make them a focal point in regenerative medicine and therapeutic applications. One of the most significant features of MSCs is their differentiation potential. These cells can differentiate into various cell types, including osteocytes, chondrocytes, and adipocytes, depending on the specific microenvironment and signaling cues they receive. This plasticity allows MSCs to contribute to tissue repair and regeneration in various organs, including the reproductive system [12].

Another critical property of MSCs is their immunomodulatory capability. They possess low immunogenicity, meaning they can evade the host immune response more effectively than other cell types. This characteristic is particularly advantageous in therapeutic settings, as it reduces the risk of rejection when MSCs are transplanted into patients. Studies have shown that MSCs can modulate both innate and adaptive immune responses by secreting various cytokines and growth factors. For instance, they can inhibit the proliferation of T cells and promote the differentiation of regulatory T cells, thereby creating an immunosuppressive environment conducive to healing and repair [13].

MSCs also exhibit anti-inflammatory properties, which are crucial for their therapeutic effects. They can secrete anti-inflammatory cytokines such as interleukin-10 (IL-10) and transforming growth factor-beta (TGF-β), which help mitigate inflammation in damaged tissues. This property is particularly relevant in conditions associated with infertility, where inflammation can hinder reproductive function [14].

Moreover, MSCs demonstrate a high capacity for self-renewal and expansion in vitro. This characteristic allows for the generation of large quantities of cells for therapeutic use without losing their functional properties. The ability to expand MSCs while maintaining their biological characteristics is essential for developing effective cell-based therapies [15].

## Types of MSCs

MSCs can be derived from various tissues, each offering unique characteristics and potential applications in regenerative medicine. The primary types of MSCs include bone marrow-derived MSCs (BMSCs), adipose-derived MSCs (ADSCs), menstrual blood-derived MSCs (MenSC), and umbilical cord-derived MSCs (UC-MSCs) [16].

BMSCs are among the most studied and widely used in clinical applications. They are isolated from the bone marrow, where they constitute a small fraction of the total cell population. BMSCs possess strong differentiation capabilities and can give rise to osteoblasts, chondrocytes, and adipocytes. Their immunomodulatory properties make them suitable for treating various conditions, including autoimmune diseases and injuries. However, the extraction process can be invasive, and the yield of viable cells is relatively low, necessitating extensive culture expansion to obtain clinically relevant quantities [17].

ADSCs are obtained from adipose tissue and have gained popularity due to their abundance and ease of extraction through minimally invasive procedures like liposuction. ADSCs exhibit similar differentiation potential as BMSCs but often show superior proliferation rates and a higher yield of stem cells per gram of tissue. Their immunosuppressive properties also make them attractive for therapeutic use in regenerative medicine, particularly in treating injuries and degenerative diseases [18].

MenSCs are a relatively novel source of stem cells that can be collected non-invasively during menstruation. These cells have shown promising potential for differentiation into various cell types while exhibiting immunomodulatory properties similar to those of BMSCs and ADSCs. Their unique origin may provide advantages in reproductive health applications, particularly concerning female infertility [19].

UC-MSCs are harvested from the Wharton’s jelly of the umbilical cord. They are considered an excellent source due to their high proliferation capacity and low immunogenicity. UC-MSCs have demonstrated significant potential in regenerative therapies due to their ability to differentiate into multiple lineages and their favorable safety profile in clinical applications. Their use is particularly appealing in pediatric medicine given their ethical sourcing [20].

## Clinical Applications of MSCs

MSCs have shown enormous potential for clinical applications due to their unique properties such as immunomodulation and potential to differentiate into multiple cell types [21]. MSCs are multipotent stem cells that can differentiate into a variety of cell types, including osteocytes, chondrocytes and adipocytes. They can be isolated from various adult tissues such as bone marrow, umbilical cord blood, adipose tissue and dental pulp. Once isolated, MSCs can be expanded efficiently in culture while maintaining their stem cell properties [9]. One of the major applications of MSCs is in cell-based regenerative therapies. Due to their ability to differentiate into cells of the musculoskeletal system, MSCs have been investigated for bone, cartilage and tendon repair [22]. In clinical trials, MSCs have been used to treat non-healing fractures [23], bone defects caused by tumors, osteonecrosis and bone defects caused by trauma [24]. Studies have also shown promising results for cartilage defects in knees [25]. The differentiation and paracrine properties of MSCs aid in cartilage regeneration and repair of defects. MSCs are also being explored for tendon injuries and regenerating tendon tissue [26]. Another important application of MSCs is in treatment of graft versus host disease (GVHD). GVHD is a common complication of allogeneic hematopoietic stem cell transplantation wherein donor T cells attack the host tissues. Due to their immunomodulatory properties, MSCs have been explored for GVHD prophylaxis and treatment. Pre-clinical and clinical studies have shown that MSCs can suppress activated T cells and inhibit immune responses, thereby preventing or treating GVHD [27]. This has led to MSCs being regarded as a promising candidate for improving outcomes in hematopoietic stem cell transplantation. MSCs also hold potential for treating neurodegenerative and neurological disorders due to their ability to migrate to injured sites and secrete neurotrophic factors [28]. Preliminary clinical trials have investigated the use of MSCs in treatment of conditions like amyotrophic lateral sclerosis (ALS) [29], multiple sclerosis (MS) [30] and stroke [31]. MSCs have been found to reduce inflammation, protect neurons from damage and promote tissue repair in the central nervous system (CNS) [31]. Further research is ongoing to evaluate their efficacy and safety for different neurological applications. The immunomodulatory properties of MSCs have also opened up possibilities for their use in treatment of autoimmune diseases [32]. Conditions like systemic lupus erythematosus (SLE), rheumatoid arthritis (RA), type 1 diabetes (T1DM) and inflammatory bowel disease (IBD) involve immune dysregulation and tissue damage. Pre-clinical models have shown that MSCs can suppress aberrant immune responses and reduce inflammation in these diseases [33]. Phase I/II clinical trials evaluating MSC therapies for autoimmune diseases have reported positive outcomes in terms of reduced disease activity and improved clinical parameters [34]. Larger controlled studies are now assessing the long-term therapeutic potential of MSCs for autoimmunity. In summary, MSCs have emerged as an attractive tool for clinical applications due to their extensive expansion potential and pleiotropic functions. Ongoing studies continue to explore innovative methods to effectively and safely deliver MSC therapies for a variety of conditions involving injuries, immune dysregulation and neurodegeneration. As the understanding of MSC biology expands, these cells hold promise to revolutionize regenerative medicine and cell-based therapies in the future.

## Application of MSCs in Female Infertility

**Table:** Table1. Effects of MSCs on Female Reproductive Diseases

**Article**	**MSC type**	**Animal Model**	**Disease**	**Effect**
				· Decrease of follicle-stimulating hormone.
Abd-Allah et al. [41]	BMSC	Rabbit	POF	· Increase of estrogen and VEGF.
				· Increase of follicle numbers with apparent normal structure of ovarian follicles.
Badawy et al. [42]	BMSC	Mice	POF	· Drop in estradiol and rise in follicle-stimulating hormone levels.
				· Presence of newly formed primordial follicles.
Fu et al. [43]	BMSC	Rat	POF	· Increase of ovarian weight and follicle counts.
				· Increase of E2 levels and decreased FSH levels.
				· Improve of the follicular morphology.
Sun et al. [63]	BMSC	Rat	POF	· Inhibition of the expression of apoptosis-related protein.
				· Repress cisplatin-induced granulosa cells apoptosis and increased cells viability.
				· Recover the estrus cycle.
Yang et al. [64]	BMSC	Rat	POF	· Increase of the number of basal and sinus follicles in POF rats.
				· Increase of E2 and AMH levels.
				· Reduction of FSH and LH levels.
Terraciano et al. [65]	ADSC	Mice	POF	· Increase of the number of follicles with apparent normal structure.
Sun et al. [37]	ADSC	Mice	POF	· Improve of ovarian function.
				· Increase of follicles at different stages and ovulation.
Su et al. [36]	ADSC	Rat	POF	· Increase of follicle counts, E2 levels and pregnancy rates.
Liu et al. [49]	MenSC	Mice	POF	· higher levels of ovarian markers.
				· Increase of ovarian weight, plasma E2 level, and the number of normal follicles.
Manshadi et al. [50]	MenSC	Rat	POF	· High plasma levels of E2 and P4.
				· Reduction of cumulus cells apoptosis.
Wang et al. [58]	UC-MSC	Mice	POF	· Recover ovary function.
				· Elevation of sex hormone.
Song et al. [56]	UC-MSC	Rat	POF	· Recover of disturbed hormone secretion and folliculogenesis.
				· Reduction of ovarian cell apoptosis.
				· Increase of proliferation of endometriotic ovarian cell.
Abomaray et al. [38]	ADSC	Cell culture	Endometriosis	· Decrease of apoptosis
				· Increase of survival of endometriotic ovarian cell.
Wang et al. [66]	MenSC	Mice	Endometriosis	· Reduction of apoptosis in granulosa cells and the fibrosis of ovarian interstitium.
				· Protective effects on damaged ovaries partially by secreting FGF2.
Gao et al. [45]	BMSC	Rat	Asherman syndrome	· Improve of reproductive outcomes.
Chan Ra et al.	ADSC	Rat	Asherman syndrome	· Regeneration of endometrium.
Domnina et al. [53]	MenSC	Rat	Asherman syndrome	· Improved of the fertility.
Wang et al. [60]	UC-MSC	Rat	Preeclampsia	· Inhibition of inflammation.
Xiong et al. [67]	UC-MSC	Rat	Preeclampsia	· Increase in the number and quality of fetuses, placenta quality, MVD and VEGF expression.
Xie et al. [68]	UC-MSC	Mice	PCOs	· Improve the pathological changes of PCOS.
				· Downregulation the expression of proinflammatory factors (TNF-α, IL-1β, and IFN-γ) and fibrosis-related genes in ovarian and uterus tissues.

**VEGF:** vascular endothelial growth factor, **MVD:** micro-vascular density, **FGF2:** fibroblast growth factor 2, **E2:** estradiol, **AMH:** anti-Mullerian hormone, **FSH:** follicle stimulating hormone, **LH:** luteinizing hormone (LH), **MSC:** mesenchymal stem cell, **BMSC:** bone marrow stem cell, **ADSC:** adipose-derived stem cell, **MenSC:** menstrual blood-derived mesenchymal stem cell, **UC-MSC:** umbilical cord mesenchymal stem cell, **POF:** premature ovarian failure, **TNF:** tumor necrosis factor, **IL:** interleukin, **PCOS:** polycystic ovary syndrome, **IFN:** interferon.

MSCs have emerged as a promising cell-based therapy for addressing infertility caused by various reproductive disorders [5]. Their regenerative and immunomodulatory properties make them suitable candidates for restoring ovarian function and repairing endometrial damage. The therapeutic potential of MSCs in infertility has been explored in conditions such as premature ovarian failure (POF), polycystic ovary syndrome (PCOS), endometriosis, Asherman syndrome, and preeclampsia (Table-1) [35]. MSCs, due to their multipotency, ability to self-renew, and lack of ethical concerns compared to other stem cell types, have been extensively investigated in preclinical models and increasingly in clinical settings [9]. This section reviews the application of MSCs from different sources in infertility treatments, highlighting their unique characteristics, mechanisms, and therapeutic potential.

ADSCs have been shown to improve ovarian function in models of POF, by enhancing follicular development and restoring hormone levels. These effects are attributed to the secretion of anti-inflammatory cytokines and growth factors, which promote tissue repair and reduce ovarian damage [36][37]. ADSCs have also demonstrated efficacy in treating endometrial damage [38].

The combination of ADSCs with scaffolds such as collagen enhances their retention and function in the damaged endometrial tissue, promoting the regeneration of the endometrial lining. This is particularly useful in conditions like Asherman syndrome, where fibrosis leads to impaired endometrial function [39].Caution is advised when using ADSCs in conditions like endometriosis, where their pro-angiogenic properties could potentially exacerbate the growth of ectopic endometrial tissue [38].

BMSCs are among the most studied MSCs for the treatment of female infertility, particularly in POF. POF is defined by the loss of ovarian function before the age of 40, often caused by chemotherapy, autoimmune disorders, or genetic factors, leading to early menopause and infertility [40].

Chemotherapy-induced ovarian failure has been one of the primaries focuses of research into MSC therapies, as conventional treatments for such conditions offer limited success. In models of chemotherapy-induced POF, BMSCs have demonstrated the ability to increase ovarian reserve, improve folliculogenesis, and reduce apoptosis in granulosa cells. These effects are mediated primarily through the secretion of bioactive factors such as vascular endothelial growth factor (VEGF) and insulin-like growth factor (IGF), which promote angiogenesis and repair of ovarian tissues [41][42][43]. In addition to ovarian regeneration, BMSCs have been found to enhance endometrial receptivity, particularly in cases of Asherman syndrome, a disorder characterized by intrauterine adhesions and fibrosis. BMSC therapy reduces fibrosis and promotes the regrowth of endometrial tissue, enhancing the chances of successful embryo implantation [44][45][46][47]. BMSC transplantation has been shown to trigger tissue repair through paracrine signaling, whereby the transplanted cells secrete cytokines, growth factors, and extracellular vesicles like exosomes. These molecules mediate anti-apoptotic and anti-inflammatory effects, which are crucial for tissue regeneration [48]. MenSCs have emerged as a promising source of MSCs for infertility treatment due to their non-invasive collection method, high proliferative capacity, and strong regenerative potential [35]. MenSCs have demonstrated the ability to restore ovarian function in POF models by increasing the number of ovarian follicles, reducing apoptosis in granulosa cells, and improving overall ovarian structure [49][50][51]. MenSCs have shown significant promise in regenerating damaged endometrial tissue, particularly in Asherman syndrome. Their ability to promote endometrial growth, vascularization, and reduce fibrosis makes them a valuable therapeutic option for enhancing endometrial receptivity and improving fertility outcomes. Furthermore, MenSCs exhibit strong immunomodulatory properties, which are critical in reducing inflammation and promoting tissue repair [52][53]. Due to ease of isolation and regenerative potential, MenSCs offer a unique advantage in the treatment of infertility [54]. Their ability to differentiate into endometrial-like cells and their high proliferative rate make them an attractive option for both preclinical and clinical applications [54].

UC-MSCs have been extensively studied in the treatment of POF and other ovarian dysfunctions, with preclinical studies showing that they promote follicular development, enhance angiogenesis, and inhibit apoptosis in ovarian cells [55][56][57]. In animal models, UC-MSCs have been shown to improve ovarian function by increasing the number of follicles and reducing granulosa cell apoptosis [58]. UC-MSCs also promote the secretion of angiogenic factors such as VEGF and hepatocyte growth factor (HGF), which support the regeneration of ovarian tissues and blood vessels, thus improving ovarian reserve and function [59]. Furthermore, UC-MSC in models of preeclampsia, where they have been shown to reduce systemic inflammation, improve placental function, and ameliorate preeclampsia symptoms [60][61][62].

## Challenges and Limitations

While MSC therapy holds promise for treating female infertility, certain challenges need to be addressed. First, optimal isolation methods, characterization and potency assessment protocols for MSCs require further standardization. The low viability and differentiation potential of MSCs after in vitro expansion poses difficulties. Second, the mechanisms of MSC action are still not fully elucidated. Moreover, their long-term fate and safety after in vivo administration requires more data. Large animal toxicity and tumorigenicity studies are needed. Third, well designed controlled clinical trials on homogeneous patient groups are still limited. Establishing efficacy parameters and assessing outcomes longitudinally presents challenges. Fourth, localizing MSC action after systemic administration can be difficult due to unclear homing signals. Effective targeted delivery methods need optimization. Lastly, high costs and regulatory hurdles currently limit scale up and accessibility of MSC therapy in clinics.

## Future Perspectives

Considerable progress has been made in understanding the regenerative properties of MSCs and translating them to preclinical models of infertility conditions. Looking ahead, a number of promising avenues need to be explored further. Larger controlled clinical trials are warranted to substantiate preliminary evidence and establish efficacy parameters. Comparative analysis of different MSC sources and doses may help optimize protocols. Combination therapies using MSCs alongside conventional treatments also require investigation. Cell priming through genetic modification or preconditioning may enhance MSC function and targeting. Novel non-invasive delivery methods combining scaffolds, biomaterials and cell carriers hold potential to effectively localize MSCs at disease sites. Establishing standardized protocols for MSC quality control shall accelerate clinical adoption. Big data and "omics" tools may help elucidate molecular mechanisms of MSC action in reproduction. Safety assessment through long term tracking of MSC fate requires further studies. Setting up biobanks and building infrastructure shall aid research reproducibility. Overall costs need to be reduced through regulatory harmonization and industrial partnerships. With ongoing innovation, MSC therapy could transform infertility management by addressing underlying pathologies through personalized regenerative approaches.

## Conclusion

In conclusion, MSCs demonstrate promising therapeutic effects in addressing several causes of female infertility through their regenerative properties. While preliminary evidence from preclinical and initial clinical studies is encouraging, further research is still warranted to fully establish the mechanisms, effectiveness, safety and technical aspects of MSC therapy. With standardization and large-scale trials, MSC-based regenerative solutions hold potential to revolutionize treatment of different forms of infertility, minimize physical burdens, and enhance success rates in the future. With judicious development, they may emerge as an alternative to or adjunct with existing fertility management options.

## Conflict of Interest

None.
